# Poly-L-Lysine-Based αGal-Glycoconjugates for Treating Anti-αGal IgE-Mediated Diseases

**DOI:** 10.3389/fimmu.2022.873019

**Published:** 2022-03-31

**Authors:** Sara Olivera-Ardid, Daniel Bello-Gil, Alexander Tuzikov, Ricardo N. Araujo, Yara Ferrero-Alves, Blanca Esther García Figueroa, Moisés Labrador-Horrillo, Ana L. García-Pérez, Nicolai Bovin, Rafael Mañez

**Affiliations:** ^1^RemAb Therapeutics, Mòdul de Recerca B, UAB Bellaterra, Barcelona, Spain; ^2^Department of Chemical Biology of Glycans and Lipids, Shemyakin-Ovchinnikov Institute of Bioorganic Chemistry Russian Academy of Sciences (RAS), Moscow, Russia; ^3^Laboratório de Artrópodes Hematófagos, Departamento de Parasitologia, ICB/UFMG, Belo Horizonte, Brazil; ^4^MEGA: Asthma Inception and Progression Mechanisms, Complejo Hospitalario de Navarra (CHN), Pamplona, Spain; ^5^Instituto de investigación sanitaria de Navarra (IdiSNA), Pamplona, Spain; ^6^ARADyAL Research Network, Instituto de Salud Carlos III (ISCIII), Madrid, Spain; ^7^Department of Medicine, Universitat Autònoma de Barcelona (UAB), Barcelona, Spain; ^8^Allergy Section, Internal Medicine Department, Hospital Universitari Vall d’Hebron (HUVH), Barcelona, Spain; ^9^Immunomediated Diseases and Innovative Therapies, Vall d’Hebron Institut de Recerca (VHIR), Barcelona, Spain; ^10^Departamento de Sanidad Animal, Instituto Vasco de Investigación de Desarrollo Agrario (NEIKER), Derio, Spain; ^11^Hospital Universitari de Bellvitge, Servicio de Medicina Intensiva, Hospitalet de Llobregat, Barcelona, Spain; ^12^Instituto de Investigación Biomédica de Bellvitge (IDIBELL), Grupo Inmunidad Innata y Patología del Paciente Crítico, Hospitalet de Llobregat, Barcelona, Spain

**Keywords:** αGal-syndrome, poly-L-lysine-based αGal-glycoconjugates, anti-αGal IgE inhibition, GalT-KO mice, immunotherapy

## Abstract

Anti-αGal IgE antibodies mediate a spreading allergic condition known as αGal-syndrome (AGS). People exposed to hard tick bites are sensitized to αGal, producing elevated levels of anti-αGal IgE, which are responsible for AGS. This work presents an immunotherapy based on polymeric αGal-glycoconjugates for potentially treating allergic disorders by selectively inhibiting anti-αGal IgE antibodies. We synthesized a set of αGal-glycoconjugates, based on poly-L-lysine of different degrees of polymerization (DP1000, DP600, and DP100), to specifically inhibit *in vitro* the anti-αGal IgE antibodies in the serum of αGal-sensitized patients (n=13). Moreover, an animal model for αGal sensitization in GalT-KO mice was developed by intradermal administration of hard tick’ salivary gland extract, mimicking the sensitization mechanism postulated in humans. The *in vitro* exposure to all polymeric glycoconjugates (5-10-20-50-100 µg/mL) mainly inhibited anti-αGal IgE and IgM isotypes, with a lower inhibition effect on the IgA and IgG, respectively. We demonstrated a differential anti-αGal isotype inhibition as a function of the length of the poly-L-lysine and the number of αGal residues exposed in the glycoconjugates. These results defined a minimum of 27 αGal residues to inhibit most of the induced anti-αGal IgE *in vitro*. Furthermore, the αGal-glycoconjugate DP1000-RA0118 (10 mg/kg sc.) showed a high capacity to remove the anti-αGal IgE antibodies (≥75% on average) induced in GalT-KO mice, together with similar inhibition for circulating anti-αGal IgG and IgM. Our study suggests the potential clinical use of poly-L-lysine-based αGal-glycoconjugates for treating allergic disorders mediated by anti-αGal IgE antibodies.

## Introduction

Type-I allergic conditions are disorders mediated by IgE, eliciting hypersensitivity to various allergens (1). IgE antibodies orchestrate an abnormal adaptive response against non-infectious, harmless, exogenous, and environmental substances, including glycoproteins from grass, pollen, dust mites, insect venom, and food (1). The number of people affected by such disorders is continuously growing globally (2, 3). Plasma exchange, immunosuppressive drugs, and monoclonal antibodies are potential immunotherapies focused on reducing IgE levels (4). Omalizumab (Xolair^®^), an unspecific treatment directed to total IgE, is the only anti-IgE therapy approved to treat moderate to severe asthma and chronic idiopathic urticaria (5). However, natural IgE has been described to participate in the physiological host resistance against certain parasites such as arthropods and helminths (6, 7). Thus, the total removal of IgE is a concern.

Increasing evidence describes the functional involvement of anti-αGal antibodies in different human disorders (8), including αGal-syndrome (AGS) (9, 10). These antibodies bind to Galα1,3Gal and Galα1,3Galβ1,4GlcNAc oligosaccharides (αGal) (11), although with higher affinity to the free trisaccharide (12). Primates, including apes, and Old-World monkeys, do not express the αGal epitopes due to an evolutive inactivation of the gene coding for the α1,3-galactosyltransferase enzyme (13, 14); consequently, they naturally produce these antibodies. Some evidence links their origin to the gut microbiota (15, 16). αGal residue is expressed in glycolipids and glycoproteins of the cell membrane of different microorganisms, including viruses, bacteria, and protozoans (8). Hence, it has been associated with a possible protective role of anti-αGal antibodies (17). However, the existing epidemiological evidence is controversial. High serological levels of anti-αGal IgM at the start of dialysis therapy have been described as a predictor of later risk for mortality and enteric peritonitis in peritoneal dialysis patients (18). Furthermore, anti-αGal IgM was associated with protection against malaria in infants (19) and children >4 years old (20). On the contrary, anti-αGal IgG has been associated with a higher risk of malaria infection in children (19, 20). Additionally, anti-αGal IgM and IgG, but not IgE antibodies, were significantly higher in uninfected than *Plasmodium falciparum*- and *Mycobacterium tuberculosis*-infected individuals (21). Similarly, experimental models have also demonstrated protection against lethal *Trypanosoma cruzi* challenge (22) and malaria by prophylactic vaccination with an αGal-based compound and oral administration of *Escherichia coli* O86:B7 (20), respectively. Interestingly, oral administration of the same bacterium protects turkeys from developing acute aspergillosis. Nevertheless, this effect was not associated with augmented anti-αGal IgY levels but with an apparent reduction of anti-αGal IgA in the lungs of infected animals (23).

**Figure 1:**
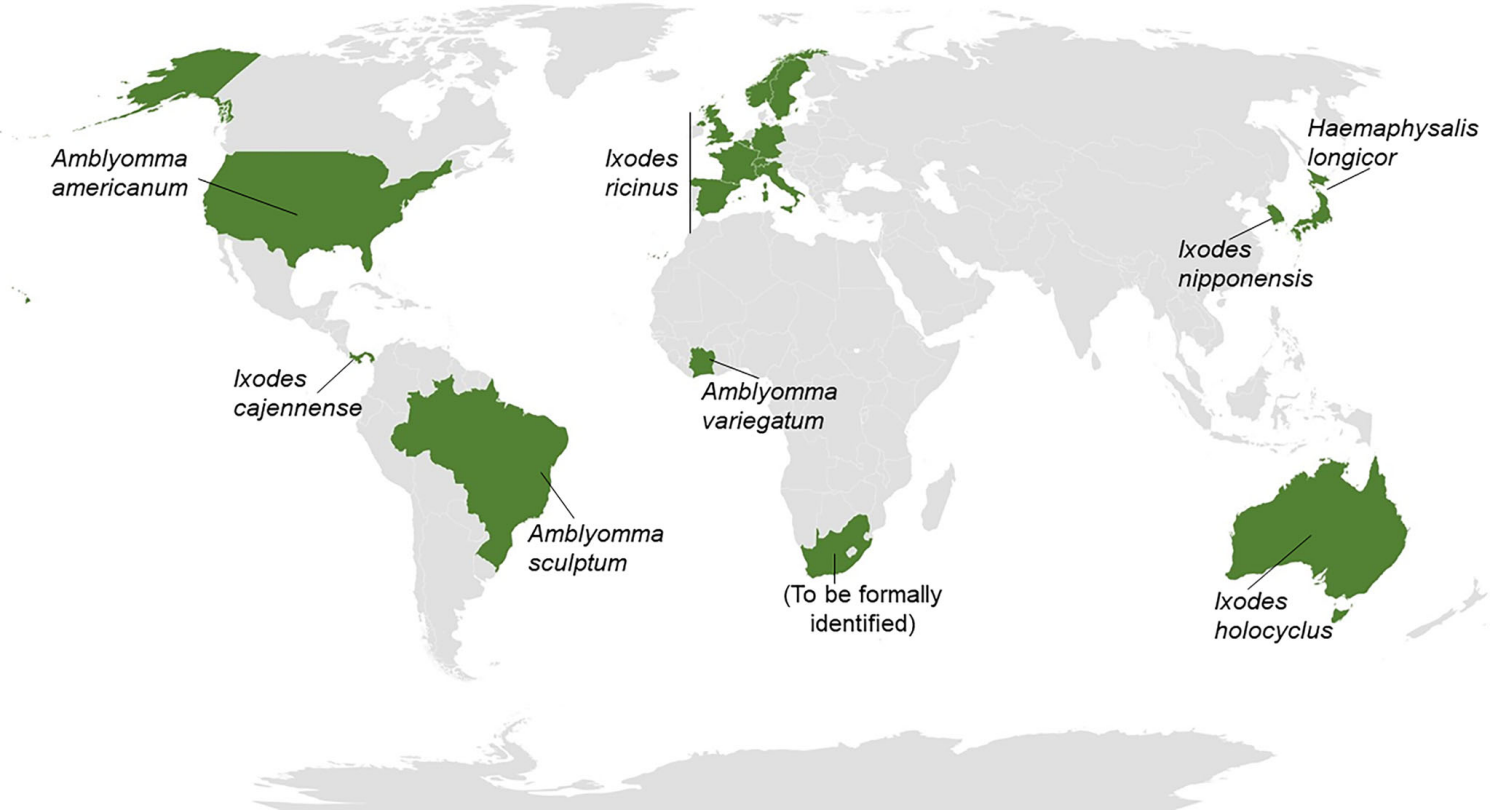
Hard ticks as the cause for αGal syndrome. Worldwide map reflecting (in green) the 17 countries in which hard ticks from different species have been detected to be causative of αGal syndrome (AGS). The list of countries includes Australia, United States, Germany, France, Spain, Belgium, Switzerland, Sweden, United Kingdom, Italy, Norway, Korea, Japan, Panama, Brazil, Ivory Coast, and South Africa.

AGS symptoms occur after red meat intake (24) or exposition to other products containing αGal like Cetuximab (25). Hard ticks are associated with the anti-αGal IgE sensitization and AGS spreading (26, 27) (**Figure 1**). The prevalence of this pathological condition is higher in countries where individuals are in contact with ticks (27). The natural habitat of this type of tick is being significantly impacted by climate change, occupying ever larger regions worldwide (28). Therefore, a notable increase in patients with AGS is expected in the coming years. Currently, the seventeen countries where there are registries of hard ticks as the causative agent of the αGal syndrome include Australia [*Ixodes holocyclus* (29)] and the United States [*Amblyomma Americanum* (30)]. In Europe, the endemic tick is *Ixodes ricinus*, found in Germany (31), France (32), Spain (33), Belgium (34), Switzerland (35), Sweden (36), United Kingdom (37), Italy (38) and Norway (39). The list of affected countries also includes Korea [*Ixodes nipponensis* (40)], Japan [*Haemaphysalis longicornis* (41)], Panama [*Ixodes cajennense* (42)], Brazil [*Amblyomma sculptum* (43)], Ivory Coast [*Amblyomma variegatum* (44)] and South Africa (45). Hard ticks’ saliva, salivary glands, and midgut contain proteins decorated with αGal residues (42, 45). Sensitization is postulated to start with the allergen uptake by APCs in the epidermis (46). Then, the sensitization mechanism promotes the class switch recombination in B cells and the subsequent secretion of IgE antibodies in the skin-draining lymph nodes (46, 47). Anti-αGal IgE-switched B cell precursor seems to be a naive non-switched B cell (48). Re-exposure to the allergen leads to the activation and degranulation of mast cells and basophils. Moreover, the allergic reaction is triggered 3-6 h after mammalian meat ingestion due to the binding of anti-αGal IgE antibodies to αGal epitopes expressed in the meat (24, 49). Regarding Cetuximab allergy, anti-αGal IgE binding to αGal residues in the monoclonal antibody induces an immediate systemic allergic reaction that can be severe enough to trigger a life-threatening anaphylactic shock (50, 51).

Patients with AGS exhibit various clinical symptoms, including urticaria, pruritus, angioedema, and systematic anaphylaxis. In addition, some patients have reported specific symptoms, such as nausea, indigestion, diarrhea, and abdominal discomfort (51, 52).

Anti-αGal antibodies (IgM, IgG) have previously been removed in rodents and primates using a poly-L-lysine-based αGal-glycoconjugate (GAS914), without side effects, minimal complement activation, and no sensitization (53). GAS914 was developed to overcome the hyperacute and acute vascular xenograft rejection in pig-to-primate transplantation primarily caused by anti-αGal antibodies (53). However, GAS914 never reached the clinic due to the participation of anti-non-αGal antibodies in the mentioned rejection mechanism (54). Additionally, the development of α1,3-galactosyltransferase gene-knockout transgenic pigs made unnecessary the clinical development of GAS914 for this indication (55, 56).

The lack of sensitization, together with the high capacity of GAS914 to inhibit anti-αGal antibodies (IgM and IgG), prompted us to study the *in vitro* and *in vivo* removal of anti-αGal IgE antibodies with a set of poly-L-lysine-based αGal-glycoconjugates as a potential treatment for diseases mediated by such antibodies.

## Materials and Methods

### Polymeric αGal-Glycoconjugates

GAS914 (Novartis Pharma AG, Basel, Switzerland) is a poly-L-lysine backbone with an average degree of polymerization (DP) of 1,000 L-lysines (DP1000) and with 23-28% of lysines derivatized with the oligosaccharide Galα1,3Galβ1,4GlcNAc- (αGal) (53). GAS914 was used as anti-αGal inhibitor control. Analysis of ^1^H-NMR allowed determining the load of αGal in GAS914 (**Figure S1**).

**Figure 2:**
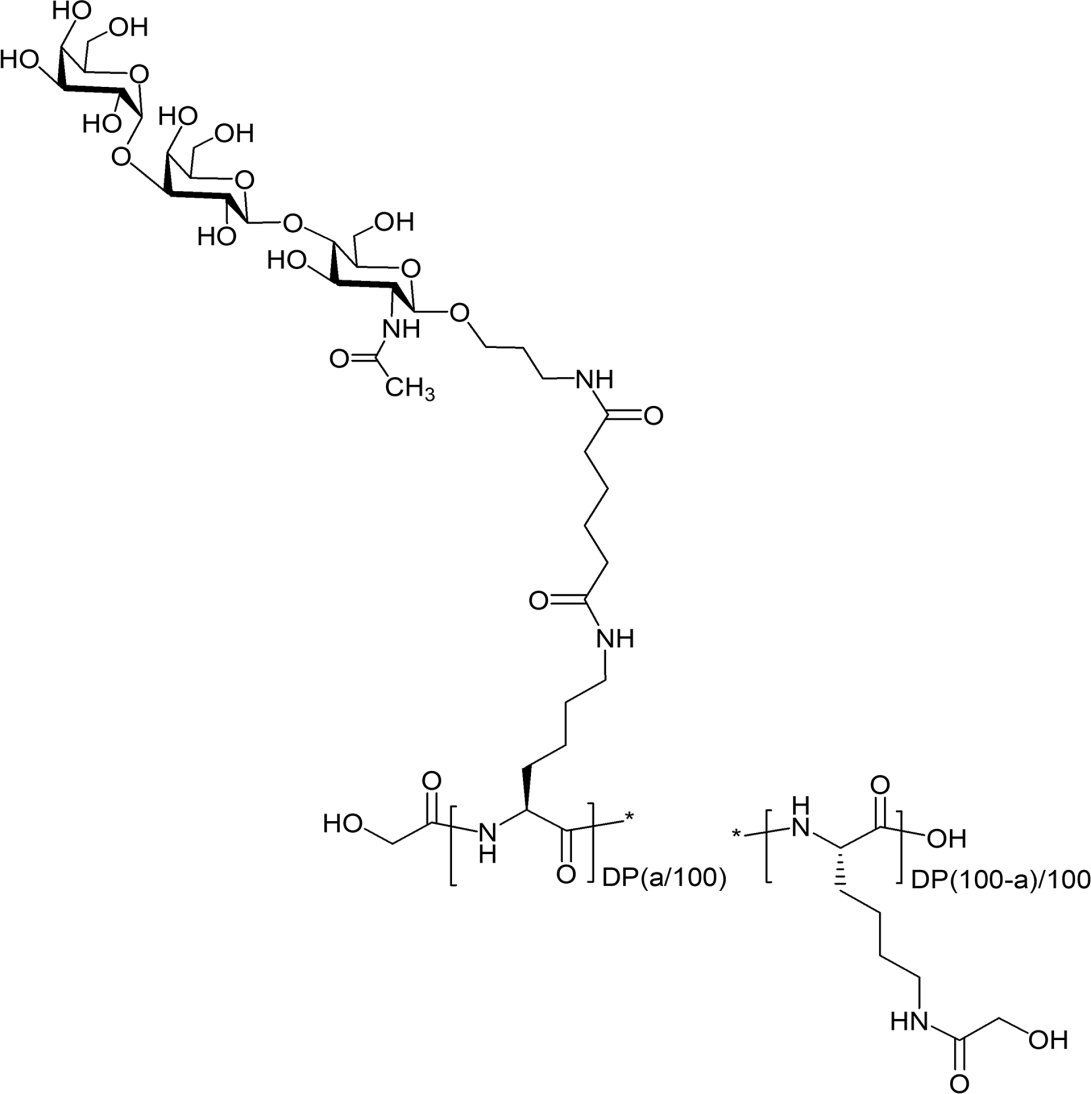
General structure of polymeric αGal-glycoconjugates. Poly-L-lysine backbone (degree of polymerization, DP: 100, 600, and 1000) were derivatized with the trisaccharide Galα1-3Galβ1-4GlcNAcβ-sp, (sp = -O(CH_2_)_3_NH_2_). Percent of modification with αGal = a (9, 12, 18, 27 and 34%). Residual amino groups of poly-L-lysine were acylated with glycolic acid.

The RA01-compounds were specifically synthesized for this work from a linear poly-L-lysine (57) of a final DP of 100, 600, 1000 (DP100, DP600, and DP1000, respectively) and increasing loads of Galα1,3Galβ1,4GlcNAc- in the final structure. DP1000 glycoconjugates were produced following the synthetic route described by Duthaler et al., 2010 (58). For DP100 and DP600 glycoconjugates, poly-L-lysine hydrobromide was acylated with a calculated amount of Galα1,3Galβ1,4GlcNAc (αGal)-sp-Ad-ONSu active ester in DMSO in the presence of Et_3_N. The residual amino groups were acylated (in the same reaction mixture) with an excess of glycolic acid acetate succinimide ester AcOCH_2_(CO)ONSu in the presence of Et_3_N. To remove acetyl protecting groups by hydrolysis, the reaction mixture was diluted with a twofold volume of water, and Et_3_N was added (2% of the volume of the solution). Glycopolymers were isolated by gel-permeating chromatography on Sephadex LH-20 in MeCN-water 30:70 by volume. Fractions contained pure conjugate were evaporated to ~2 mL volume and freeze-dried. The purity and composition of the synthesized glycoconjugates (the percentage of modification of poly-L-lysine with Galα1,3Galβ1,4GlcNAc) were determined by the ^1^H NMR spectroscopy (**Figure S2**). The complete list of resulting polymeric glycoconjugates is shown in **Table S1**. **Figure 2** provides the general structure for this set of glycopolymers.

### Human Serum Samples

Normal Human Sera (NHS) from donors of the Blood Bank of the Hospital Universitari de Bellvitge were used as healthy controls (CN, n=8). Serum samples from αGal-sensitized subjects (PT, n=13) were selected from previous epidemiological studies (59), where anti-αGal IgE prevalence in individuals with acute urticaria or anaphylaxis from different geographical areas of Spain was studied.

### *In Vitro* Inhibition of Anti-αGal With Polymeric αGal-Glycoconjugates

To test the inhibition of anti-αGal antibodies, human serum samples (PT, n=13) were incubated for 15-17 h under mild orbital shaking (225 rpm at 4°C) with each glycopolymer at growing concentrations (5-10-20-50-100 µg/mL). The final volume of the reaction was set at 100 µL. Vehicle (PBS)-treated serum was incubated under the same conditions as a control (baseline). After incubation, the serum samples containing the different glycopolymers or PBS (control for each serum) were conveniently diluted to determine the unbound fraction of the different anti-αGal isotypes, by ELISA, following the general protocol previously described (15). Briefly, Nunc MaxiSorp^TM^ 96-well flat-bottom plates (Thermo Fisher Scientific, Waltham, MA, USA) were coated with 2.5 μg/mL of Galα1,3Galβ1,4GlcNAc glycan conjugated to human serum albumin (HSA). After washing and blocking steps, serum samples diluted in PBS (1:100 for IgM and IgG, 1:25 for IgA, and 1:10 for IgE) were added to the wells and incubated for 1 h at 25°C. After washing, the incubated for 1 h at 25°C with the corresponding horseradish peroxidase (HRP)-labeled anti-human or anti-mouse secondary antibodies diluted in PBS. o-Phenylenediamine dihydrochloride (OPD) was used as HRP substrate, and incubated at 25°C in the dark. The reaction was stopped with 3N hydrochloric acid (HCl). The resulting absorbance was registered at 492 nm using a PowerWave™ XS Microplate Reader (Biotek, Winooski, VT, USA). The resulting data were graphed as optical density units. Moreover, the *in vitro* data was the result of three independent experiments. The inhibition rate for each glycopolymer (expressed as a percentage of anti-αGal inhibition) was calculated according to the levels of anti-αGal antibodies determined in baseline (PBS) and treated (glycopolymer) conditions for each serum.

### α1,3-Galactosyltransferase Knocked Out (GalT-KO) Mice

This study was performed in 48 mice of 24-32 weeks-old (sex parity), in which the gene coding for the α1,3-galactosyltransferase enzyme had been knocked out (GalT-KO mice) and was derived from a highly inbred colony with a hybrid genetic background (B6xCBAx129sv) (60). Animals were handled and housed as previously described (15). Procedures concerning all animals were supervised and approved by the ethics committee for animal experimentation of Bellvitge Biomedical Research Institute (IDIBELL) and the Catalonia Government (Record FUE-2018-00931758). The care, as well as the handling of the animals, were following the Guide for the Care and Use of Laboratory Animals that the US National Institutes of Health published (NIH Publication n° 85–23 revised 1996) as well as the European Agreement of Vertebrate Animal Protection for Experimental Use (86/609). The procedure for euthanasia was established following the European Directive on protecting animals used for scientific purposes (2010/63/EU). Death was never considered a human endpoint.

### *Amblyomma sculptum* Salivary Gland Extract

As previously described (61), salivary gland extract (SGE) was produced from 200 females of unfed *A. sculptum*. To obtain the SGE, females were washed with sterile water, and their salivary glands were individually dissected in saline (0.9% NaCl). Each pair of glands were transferred to 1.5 mL tubes containing saline solution, placed in an ultrasonic bath for 40 seconds, centrifuged at 14,000*g* for 5 min. Next, the supernatant was transferred to a new tube, dried under vacuum at 56°C to yield an amount of 3.11 mg, and kept at -20°C until use. The amount of protein in the sample was measured by Bradford et al., 1976 (62) using bovine serum albumin as standard. Sterile PBS was conveniently used as a vehicle to prepare the final aqueous solution injected as an allergen to the GalT-KO mice.

### GalT-KO Mice Sensitization

**Figure 3:**
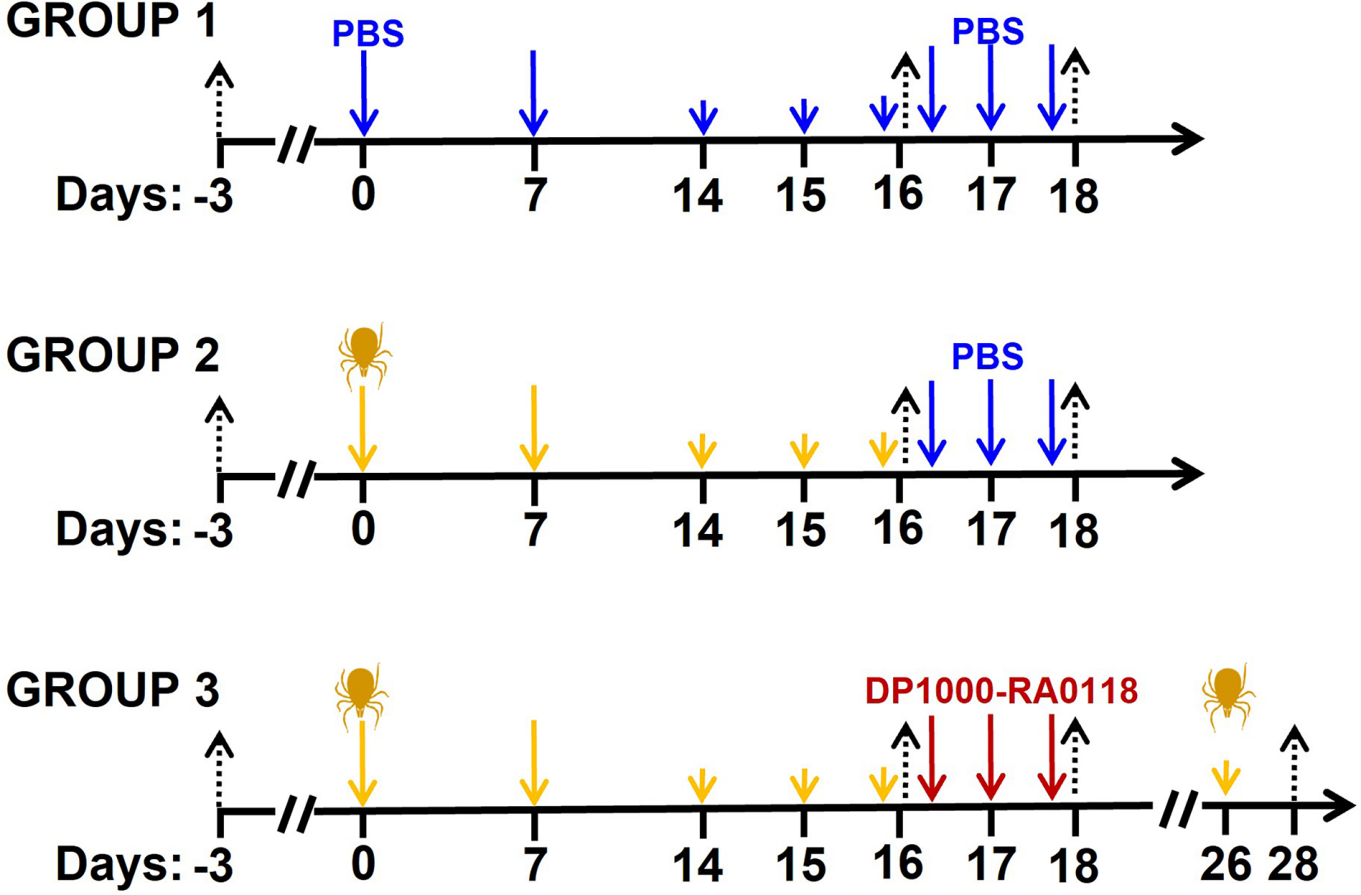
Scheme of GalT-KO mice sensitization for anti-αGal IgE production. Group 1: double negative control (PBS) (n=16). Group 2 (n=16) and Group 3 (n=16) (αGal-sensitized mice) were treated with PBS (control) and DP1000-RA0118, respectively. Black arrows: bleeding (for immunological determinations), blue arrows: PBS id. (control of sensitization) or sc. (control of treatment), yellow arrows: salivary gland extract (20 or 5 µg id.), dark-red arrows: DP1000-RA0118 10 mg/kg sc.

GalT-KO mice were randomly separated into three different groups (sex parity). Group 1 (n=16) was a double negative control (PBS id. or sc. for sensitization and treatment, respectively). Group 2 (n=16) and 3 (n=16) were sensitized with two doses of 20 μg id. of the salivary gland extract (days 0 and 7, **Figure 3**). Animals were then challenged with three consecutive doses of 5 μg id. of the extract on days 14, 15, and 16 to induce the production of anti-αGal IgE antibodies (63, 64). Group 2 was a negative control for the treatment (vehicle: PBS sc.). In Group 3, animals were treated with three consecutive DP1000-RA0118 doses (10 mg/kg, sc.) on days 16 (4 h after last challenge), 17, and 18. The challenge was repeated in two animals of Group 3 one week after the last treatment with DP1000-RA0118 (day 26). Although each experimental group was composed of sixteen animals, not all the parameters were determined in the totality of mice due to welfare reasons. In the case of multiple blood extractions, volume never exceeded 7.5% of the total blood volume (124-158 µL of fresh blood in ~30 g mice) weekly (65). Animal blood was collected by controlled submandibular bleeding on days -3 (baseline), 16 (3 h after challenge), 18 (3 h after treatment), and 28 (after rechallenging, two animals of Group 3) as previously described (66).

### Total Circulating Mouse IgM, IgG1, IgG2a, IgG2b, IgG3, and IgE by ELISA

Total mouse serum immunoglobulins (n=6) were determined on days -3, 16, and 18 using the commercial RayBio^®^ mouse ELISA kit following manufacturer instructions (RayBiotech, GA, USA).

### White Blood Cells by Flow Cytometry

Flow cytometry analysis was performed on a Gallios analyzer (Beckman Coulter, IN, USA) equipped with violet (405 nm), blue (488 nm), and red (633 nm) solid-state lasers as an excitation source. Purified rat anti-mouse CD16/CD32 was added to the fresh blood samples to block non-antigen-specific binding (Beckton Dickinson, CA, USA). After 5 min incubation at 25°C, fluorochrome-conjugated antibodies (**Table S2**, Beckton Dickinson, CA, USA) were added to the samples. BD FACS™ lysing solution was then added to lyse red blood cells. Samples were homogenized with vortex and incubated for 5 min at 25°C in the dark. After centrifugation (5 min, 3,220*g*, 25°C), the supernatant was discarded, and the pellet was resuspended in 400 µL of PBS for immunophenotyping of different White Blood Cells (WBC) for every experimental group (n=6) on days -3, 16, and 18. Events collected from fresh blood mouse samples were displayed in a CD45 vs. side scatter intensity (SS INT) plot to discard debris and define a total WBC population. Every single FACS determination recorded about 150,000 total events, of which 50,000 were CD45 positive. Fluorescence was collected through the corresponding bandpass filters for each indicated surface cell marker. Data were analyzed using KALUZA software (Beckman Coulter, CA, USA).

### Statistics

GraphPad Prism statics software was used for analysis and data graphing. The Gaussian distribution of data was checked by the D’Agostino-Pearson omnibus normality test (alpha = 0.05), and homogeneity of variances was determined by the F test (alpha = 0.05). Most of the statistical analyses were performed using paired or unpaired parametric t-tests. The Wilcoxon matched-pairs signed-rank and Mann-Whitney tests (unpaired data analysis) were used as non-parametric tests when data did not follow a Gaussian distribution. Tukey and Sidak were used as multiple comparison tests. Differences were considered statistically significant when p<0.05 (*: p<0.05; **: p<0.01; ***: p<0.001; ****: p<0.0001), ns: non-significant.

## Results

### Prevalence of Anti-αGal IgE in αGal-Sensitized Subjects

**Figure 4:**
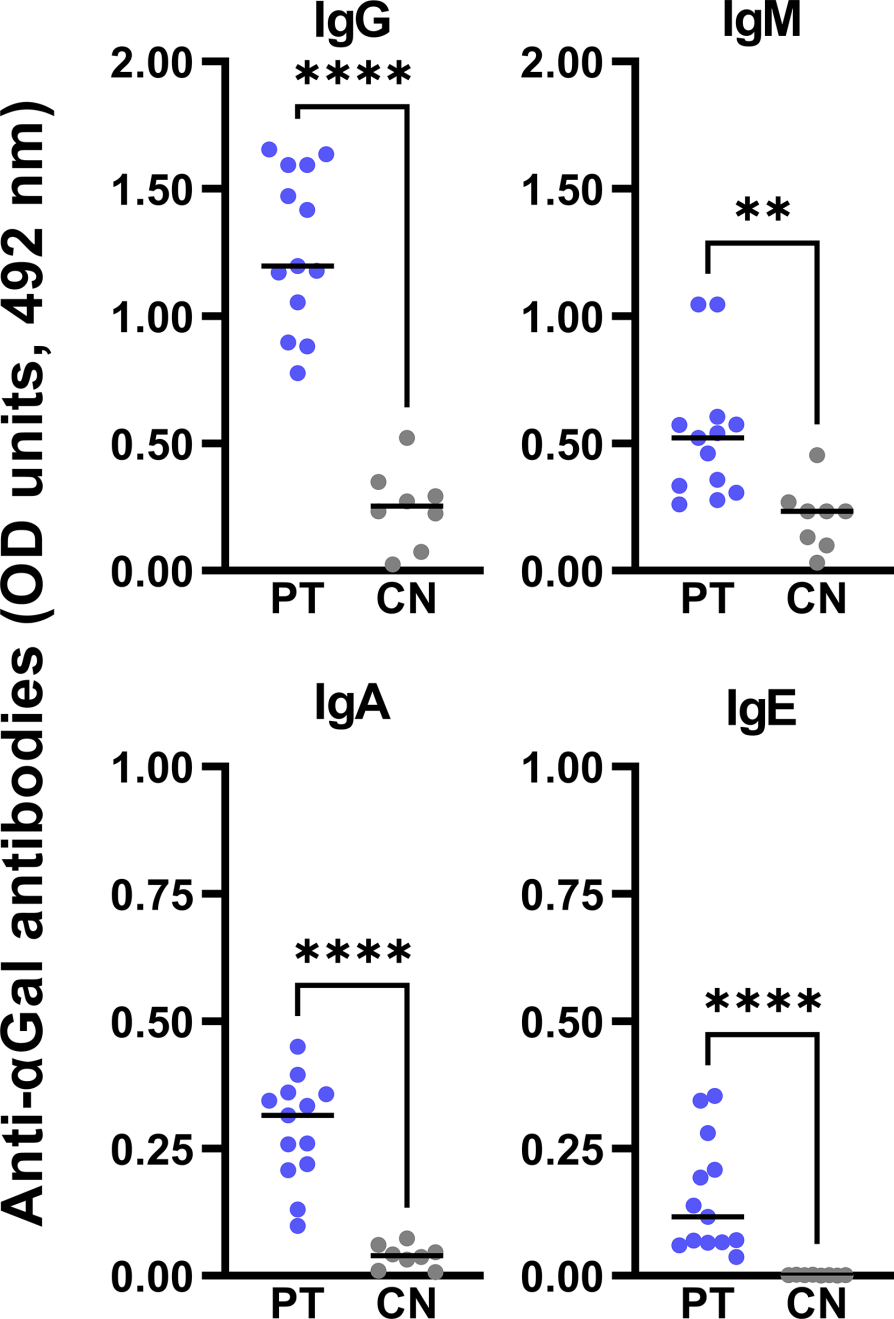
Serological levels of anti-αGal antibodies by ELISA. αGal-sensitized patients (PT, n=13, blue dots) showed significantly higher serological anti-αGal antibodies levels (expressed as optical density units, 492 nm) compared to healthy subjects (CN, n=8, grey dots). To detect each immunoglobulin isotype, serum samples were accordingly diluted: 1:100 for IgM and IgG, 1:25 for IgA, and 1:10 for IgE. Unpaired t-test analysis was performed (**: p < 0.01; ****: p < 0.0001).

The prevalence of anti-αGal IgE in αGal-sensitized subjects has been extensively reviewed (67–69). We confirmed by ELISA the significantly elevated circulating levels of anti-αGal IgE antibodies in patients compared to controls. PT also showed higher serological levels for the rest of the anti-αGal isotypes (IgM, IgG, and IgA) than CN (**Figure 4**).

### Influence of the Multivalent αGal Exposure on the Inhibition of Anti-αGal IgE Antibodies

**Figure 5:**
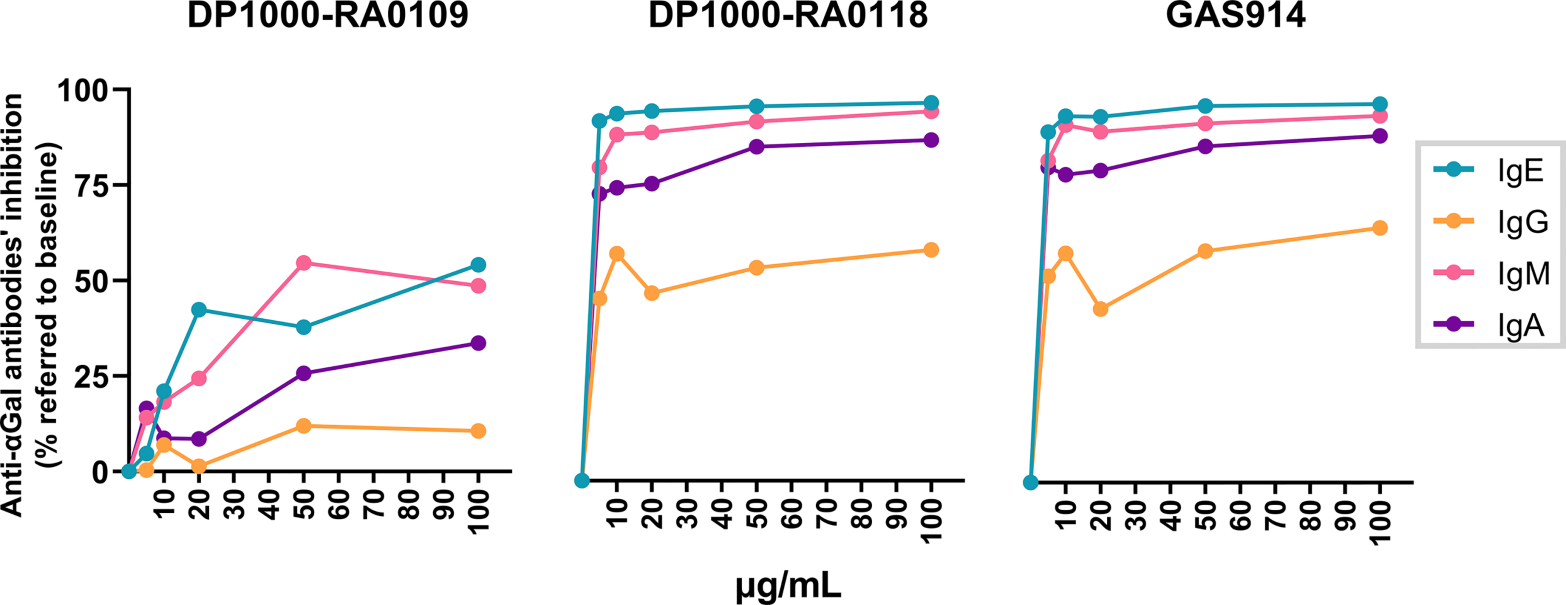
Influence of αGal load on anti-αGal antibodies inhibition. Serum samples from αGal-sensitized patients (n=13) were incubated with DP1000-RA0109, DP1000-RA0118 or GAS914 (positive control of inhibition) at growing concentrations (5-10-20-50-100 µg/mL). PBS-treated serum was similarly incubated as a baseline condition. After incubation, samples were conveniently diluted to determine by ELISA the unbound fraction of the different anti-αGal isotypes (1:100 for IgM and IgG, 1:25 for IgA, and 1:10 for IgE). The inhibition rate for each glycopolymer (expressed as a percentage of anti-αGal inhibition) was calculated according to the quantity of anti-αGal antibodies determined in baseline (PBS) and treated (glycopolymer) conditions for each serum. IgE green line, IgG orange line, IgM pink line, and IgA purple line.

Anti-αGal antibodies inhibition with monovalent compounds has been demonstrated as inefficient due to their low affinity for single oligosaccharides (53). Previously, GAS914 has shown a maximal increase in avidity (relative to the monomer) by anti-αGal IgM and IgG antibodies (53, 70). Nevertheless, there is no data on whether the anti-αGal IgE inhibition could be affected using multivalent αGal compounds. For that, we synthesized two glycopolymers composed of a backbone of 1,000 L-lysines (DP1000), with 9 and 18% of lysine residues derivatized with Galα1,3Galβ1,4GlcNAc- (DP1000-RA0109 and DP1000-RA0118, respectively). GAS914 (DP1000) with 27% αGal load (**Figure 5**) was used as a positive control for anti-αGal inhibition.

It was previously demonstrated that the chromatographic affinity purification process could favor the preferential binding and elution of IgM over the rest of anti-αGal isotypes masking the real inhibitory capacity of polyacrylamide-based αGal-conjugates (71). Therefore, we used in the *in vitro* inhibitory studies human serum from αGal-sensitized patients instead of affinity-purified fractions of anti-αGal antibodies.

Overall, the exposure to all polymeric glycoconjugates mainly inhibited anti-αGal IgE and IgM isotypes, with a lower inhibition effect on the IgA and IgG isotypes, respectively (**Figure 5**). We found very similar inhibition rates for all the anti-αGal isotypes when comparing DP1000-RA0118 vs. GAS914 (positive control). Indeed, for IgE, DP1000-RA0118 showed slightly higher inhibition rates than GAS914. Additionally, reducing the αGal load in the same poly-L-lysine backbone to 9% (DP1000-RA0109) significantly reduced the inhibitory capacity of the glycopolymer (**Figure 5**). Hence, DP1000-RA0118 was selected for the *in vivo* proof of concept.

### *In Vivo* Inhibition of Anti-αGal IgE in Sensitized Mice

The preliminary data obtained *in vitro* prompted us to investigate, as proof of concept, the anti-αGal IgE inhibitory capacity of DP1000-RA0118 in a small animal model of αGal sensitization. Therefore, the main objective of this study was to induce anti-αGal IgE in a relevant animal model and to study its intracorporeal removal with DP1000-RA0118.

**Figure 6:**
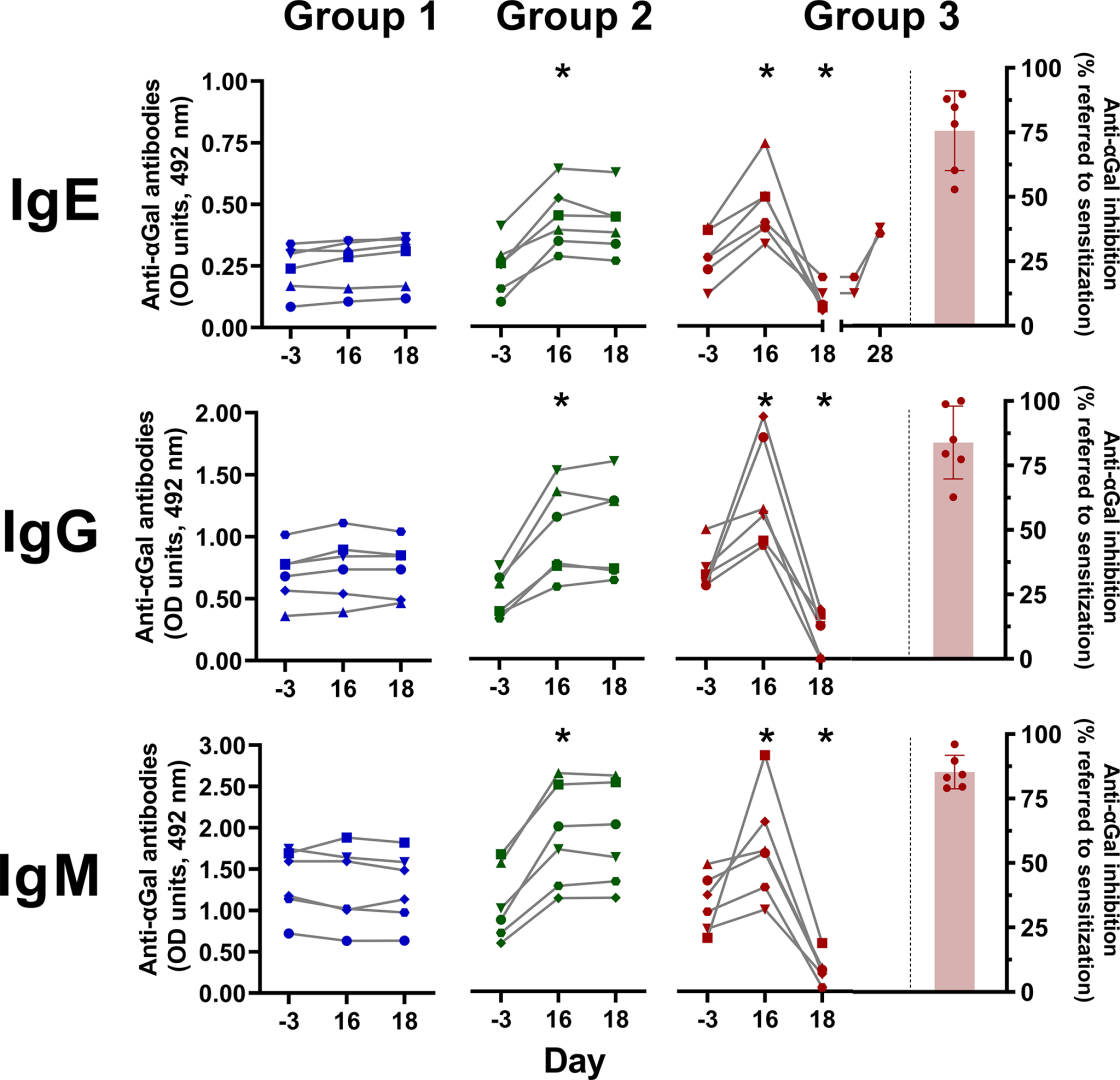
*A. sculptum* salivary gland extract significantly induced anti-αGal antibodies in GalT-KO mice. Animals from Group 1 (in blue, n=6) are a double negative control (PBS for sensitization and treatment, respectively). Animals of Group 2 (in green, n=6) and 3 (in dark-red n=6) were sensitized to αGal according to **Figure 2**. In Group 3, after sensitization, animals were treated with three consecutive DP1000-RA0118 doses (10 mg/kg, sc.) on days 16, 17, and 18. The challenge was repeated in two animals of Group 3 on day 26, one week after the last treatment with DP1000-RA0118. Red columns represent the anti-αGal inhibition on day 18 (% referred to sensitization on day 16) in Group 3 (treated with DP1000-RA0118). Wilcoxon matched-pairs signed-rank was used as a non-parametric test (*: p < 0.05).

GalT-KO mice are considered an adequate model for αGal sensitization and production of anti-αGal IgE antibodies (15, 61, 72, 73). The sensitization (**Figure 3**) was conducted in immunologically mature GalT-KO mice (15). Significant induction of anti-αGal IgE antibodies was achieved in all the animals of Groups 2 and 3 on day 16 (**Figure 6**). This induction was also accompanied by significantly augmented anti-αGal IgG and IgM. Elevated levels of anti-αGal IgE antibodies were again induced on day 28 in two mice of Group 3 rechallenged with the SGE on day 26 (**Figure 6**). The treatment with DP1000-RA018 (day 18) produced a significant decrease in the levels of anti-αGal IgE in Group 3 (≥75% on average, **Figure 6**) as well as in anti-αGal IgG and IgM compared to the induced condition (Group 3, day 16). This decrease in the levels of anti-αGal can only be attributed to the *in vivo* inhibitory capacity of DP1000-RA0118 because in Group 2 (treated with PBS) the levels of these antibodies remained constant from day 16 (end of challenge) to day 18 (end of PBS treatment).

### Impact of Sensitization and Treatment With DP1000-RA0118 on Humoral and Cellular Immune Mediators

Anti-αGal antibodies (IgM, IgG) have previously been removed in rodents and primates using GAS914 (53). Since these studies were conducted in healthy animals (no sensitized), we investigated whether some of the humoral and cellular mediators of the immune system were affected by the sensitization protocol and the treatment with DP1000-RA0118.

**Figure 7:**
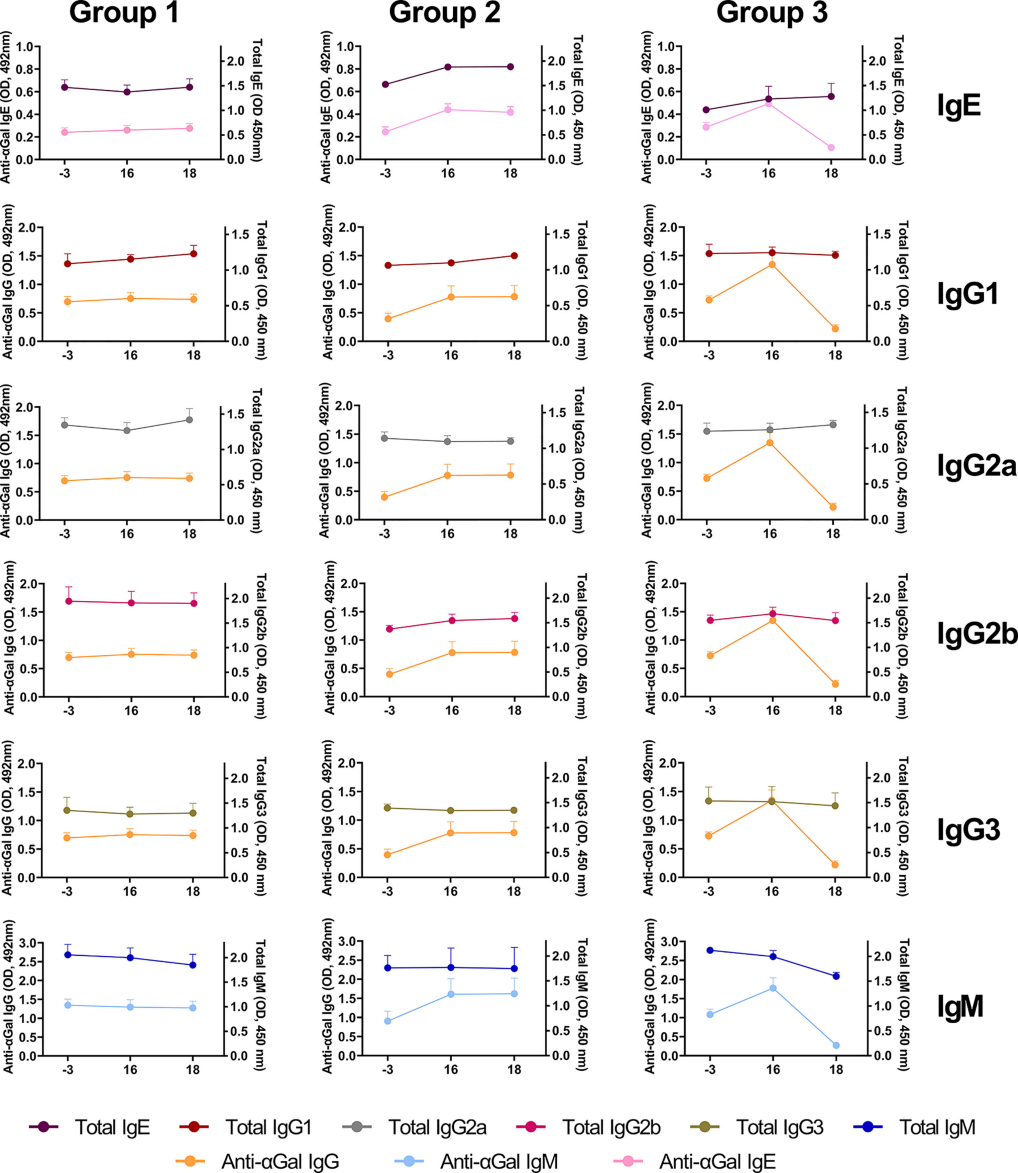
The pattern of total immunoglobulin levels (IgE, IgG1, IgG2a, IgG2b, IgG3, and IgM) and their association with the different isotypes assessed for anti-αGal antibodies (IgE, IgG, and IgM). Relative serological levels of total immunoglobulins (expressed as OD units, 450 nm) and anti-αGal antibodies (expressed as OD units, 492 nm) were determined by ELISA on days -3 (baseline), 16 (after sensitization), and 18 (after treatment). GalT-KO mice from Group 1 are a double negative control (PBS for sensitization and treatment, respectively) (n=6). Animals of Groups 2 and 3 were sensitized to αGal, according to **Figure 2**. In Group 3, after sensitization, animals were treated with three consecutive DP1000-RA0118 doses (10 mg/kg, sc.) on days 16, 17, and 18.

Regarding immunoglobulins, despite the increase obtained for anti-αGal antibodies after sensitization for Groups 2 and 3, the total IgM, IgG1, IgG2a, IgG3 remained constant during the evaluated days for all the experimental groups (**Figure 7**). However, total IgM slightly decreased from day 16 to 18 only in Group 3. The treatment with DP1000-RA0118 (day 18) removed preexisting and induced anti-αGal IgM, which impacted the total IgM levels measured. Interestingly, a slight increase in total IgE and IgG2b subtype was observed for Groups 2 and 3 after mice sensitization (day 16, **Figure 7**). These trends in both experimental groups seem to be partially associated with the induction on day 16 of augmented levels of anti-αGal antibodies for the IgE and IgG isotype, respectively.

During αGal sensitization, the major change in the WBC population was registered for basophils (**Figure S3**). The rest of the evaluated WBC remained unchanged between days and experimental groups. Basophils were gated using rat anti-mouse Ly6G and IgE (**Figure S3A**). The later monoclonal antibody allowed quantifying the IgE bound to Fcϵ receptors on basophils. On day 16, Groups 2 and 3 showed a significant increase in fluorescence compared to day -3 (baseline) (**Figures S3B, C**). Since the gated basophil population between experimental groups was similar, the augmented fluorescence obtained for Groups 2 and 3 seems to be associated with the increased serological levels of anti-αGal IgE antibodies after the sensitization procedure. On day 18, the basophil fluorescence returned to baseline levels (**Figure S3C**).

### Influence of Degree of Polymerization on the Differential Inhibition of Anti-αGal Isotypes

**Figure 8:**
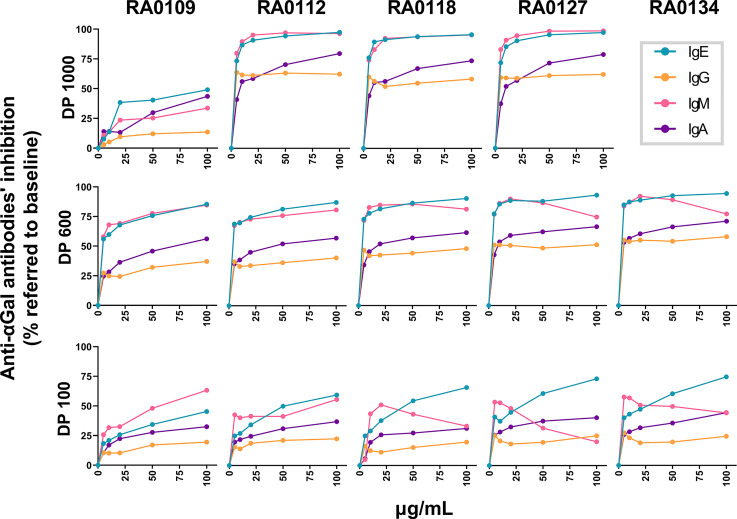
Differential antibody inhibitory capacity of αGal glycoconjugates with different DP and αGal loads in serum from αGal-sensitized patients. Serum samples (n=13) were incubated with the glycopolymers at growing concentrations (5-10-20-50-100 µg/mL). PBS-treated serum was similarly incubated as a baseline condition. After incubation, samples were conveniently diluted to determine by ELISA the unbound fraction of the different anti-αGal isotypes (1:100 for IgM and IgG, 1:25 for IgA, and 1:10 for IgE). The inhibition rate for each glycopolymer (expressed as a percentage of anti-αGal inhibition) was calculated according to the quantity of anti-αGal antibodies determined in baseline (PBS) and treated (glycopolymer) conditions for each serum. IgE green line, IgG orange line, IgM pink line, and IgA purple line.

Along with specific inhibition of anti-αGal IgE, DP1000-RA0118 showed a high inhibitory capacity to the rest of circulating anti-αGal isotypes. Although removing most of the circulating anti-αGal isotypes might not represent an obstacle to the potential clinical development of DP1000-RA0118 (74), we investigated whether the DP can impact the differential inhibition of anti-αGal isotypes. For that, we synthesized a complementary set of αGal-glycoconjugates using polymeric backbones containing 600 (DP600) and 100 (DP100) L-lysine residues. In addition, DP1000 αGal-glycoconjugates with 12% and 27% Galα1,3Galβ1,4GlcNAc load were also synthesized. All glycopolymers were assessed in the same experimental setting to avoid biases. Like the pilot study, the exposure to all polymeric glycoconjugates mainly inhibited anti-αGal IgE and IgM isotypes, with a lower inhibition effect on the IgA and IgG isotypes, respectively (**Figure 8**).

#### DP1000-Glycopolymers

From DP1000-RA0112 to DP1000-RA0127, glycoconjugates showed a similar inhibitory capacity for all isotypes at the assessed concentrations. Starting at 10 µg/mL, the inhibition was >85% for IgE and IgM, ~60% for IgG, and 50-80% for IgA. In contrast, the inhibition for DP1000-RA0109 was significantly lower than the other glycopolymers for all Ig-isotypes (**Figure 8**).

#### DP600-Glycopolymers

From DP600-RA0112 to DP600-RA0134, glycoconjugates showed a similar inhibitory capacity for all isotypes. From 10 µg/mL, the inhibition was >70% for IgE and IgM, 33-60% for IgG, and 40-72% for IgA. Surprisingly, IgM inhibition rates slightly decreased from 50 µg/mL in the case of RA0118, RA0127, and RA0134. DP600-RA0109 showed a similar trend but with lower inhibition rates. Regardless of the glycan load, all the glycopolymers inhibited >85% of circulating anti-αGal IgE at 100 µg/mL (**Figure 8**).

#### DP100-Glycopolymers

From DP100-RA0112, all DP100 glycoconjugates showed similar inhibitory capacity regardless of the Galα1,3Galβ1,4GlcNAc percentage. Starting at 5 µg/mL, the inhibition was 25-75% for IgE, 40-56% for IgM, 12-25% for IgG, and 20-45% for IgA. Similar to DP600, IgM inhibition rates decrease from 20 µg/mL in the case of RA0118, RA0127, and RA0134. To note, DP100-RA0127 and DP100-RA0134 (100 µg/mL) showed a high IgE-inhibition rate (>75%), with minimal IgG inhibition (<25%, **Figure 8**).

Interestingly, RA0112, RA0118, and RA0127 (DP600) showed similar IgE and IgA inhibitory capacities compared to DP1000 glycopolymers with the same αGal load, except for RA0112 at 10 µg/mL for IgE. Additionally, RA0118 and RA0127 (DP600) showed similar IgM and IgG inhibitory capacity compared to DP1000-homologues.

## Discussion

This work shows that immunotherapy based on the intracorporeal inhibition of anti-αGal IgE antibodies with poly-L-lysine-based αGal-glycoconjugates may be a potential solution to treat AGS. The rationale for using poly-L-lysine-based αGal-glycoconjugates on removing anti-αGal antibodies lies in: i) anti-αGal antibodies have naturally high avidity for multivalent antigens, ii) absence of immune response against either the carbohydrate or the poly-L-lysine in different murine and primate species that spontaneously produce the anti-αGal antibodies (53), and iii) anti-AB0 group antibodies (similar in origin and structure to anti-αGal antibodies) have been safely maintained at low concentrations (plasmapheresis plus immunosuppression) for a prolonged time in blood type-incompatible kidney transplantation without side effects (74).

Firstly, elevated levels of anti-αGal IgE antibodies were detected in patients compared to healthy volunteers (67–69). The same trend was observed for the rest of the anti-αGal isotypes. In the initial pilot study, we demonstrated the feasibility of improving the inhibitory capacity of αGal-glycopolymers by reducing the αGal load. This finding was unexpected according to the differences in αGal density in DP1000-RA0118 (180 residues) and GAS914 (270 residues), respectively. An unfavorable spatial conformation due to a higher αGal density in GAS914 compared to DP1000-RA0118 could explain these results (sterical hindrances) (71, 75). Conversely, DP1000-RA0109 (90 residues) showed a drastically reduced capacity to inhibit anti-αGal antibodies. The limited exposure of the antigenic determinant (antigen/antibody ratio) could explain this finding.

Additionally, anti-αGal IgE was the most inhibited isotype *in vitro* with similar behavior for IgM and a lower inhibition for IgA and IgG, respectively. The later isotypes typically have higher affinities for protein antigens (single binding site) than IgM. However, IgM is a pentamer with ten Fab capable of a multivalent binding (76). In addition, 20% of serological IgA exists as oligomers with multi-binding sites. On the other hand, IgG only exists as a monomer with two binding sites (71). These properties of antibodies were confirmed in our *in vitro* study, where the IgM avidity was increased, exceeding the affinity of IgA and IgG. Similar results have been reported for polyacrylamide-based αGal-glycoconjugates (71). However, the behavior of anti-αGal IgE, similar to IgM, was unexpected since it is a monomer like IgG (77). Despite some structural differences (78), IgE and IgG share the highest homology compared to other isotypes (78, 79). However, the inhibition of anti-αGal IgG in patients was lower compared to IgE. This finding could be explained by the fact that anti-αGal IgE are strictly induced antibodies, whereas the circulating anti-αGal IgG are mainly composed of natural antibodies. Thus, despite the relatively lower levels of anti-αGal IgE, their affinity seems to be higher than anti-αGal IgG. Consequently, we hypothesize that anti-αGal IgE might come from IgG-switched B cells, unlike the classic atopic sensitization to pollen and mite allergens that come from naive B cells (48). In contrast, previous studies described that anti-αGal IgE might be predominantly formed by class switch from non-switched (IgM) B cells (80).

We also developed a model of αGal-sensitization that reproduced the findings obtained for patients. We achieved a clear induction of anti-αGal IgE, together with IgM and IgG after intradermal administration of hard ticks’ SGE. Unlike natural anti-αGal antibodies (15), anti-αGal IgE are induced after processing the αGal residues in tick saliva by APCs (Langerhans and dermal Dendritic Cells). APCs migrate then to skin draining lymph-node where αGal-specific B cells undergo clonal selection (46). The increased levels of anti-αGal antibodies in sensitized animals confirmed the implication of components from *A. sculptum* SGE in the induction of anti-αGal antibodies (43, 81).

Furthermore, DP1000-RA0118 showed high efficacy for the intracorporeal removal of anti-αGal in GalT-KO mice. At the cellular level, the WBC population remained unaltered except for basophils that showed a higher fluorescence after the sensitization process in Groups 2 and 3 because of an augmented IgE binding to Fcϵ receptors. In humans, basophil activation can determine the severity of the clinical picture in patients with delayed anaphylaxis due to the consumption of red meat (82, 83).

The immune stimulation with the SGE was not restricted to IgE. Elevated titers of anti-αGal IgG antibodies have been previously described in IgE-positive subjects (67, 80). Specifically, the authors found more anti-αGal IgG1 antibodies in IgE-positive subjects, contrasting the expected more IgG2 production specific for natural anti-αGal antibodies (67). These results suggest that IgE directed to a carbohydrate antigen results from the stimulation of a glycoprotein or glycolipid, even a bacterial immune stimulation with essentially the same antigen already exists (80). In our study, the increase of anti-αGal IgG and IgE directly impacted the rise of total IgG2b and IgE immunoglobulins, respectively.

Additionally, high levels of anti-αGal IgE antibodies in αGal-sensitized patients have been directly correlated to a total IgE antibody increase (9). However, the significant contribution of anti-αGal IgG to the total IgG2b was surprising, indicating a preferential induction of this IgG subclass. GalT-KO mice naturally produce anti-αGal antibodies (15, 70), with IgG3 as the predominant IgG subclass (manuscript submitted to publication). In mice, the IgG3 subclass is functionally equivalent to human IgG2, which predominantly recognizes carbohydrate epitopes (84, 85). Accordingly, two immune responses could be distinguished against the αGal epitope in the animal model: (I) the typical T-independent response promoted by the continuous antigenic stimulation of the intestinal microbiota (15), with IgG3 as the predominant subclass and (II) an “atypical” Th2-response (hypersensitivity type-I) which leads to the production of IgG2b and IgE in GalT-KO mice. This immunological response is similar to that described in humans sensitized after tick bites (9, 80) and in GalT-KO models (46, 86) confirming the robustness of our animal model for αGal sensitization.

DP1000-RA0118 inhibited anti-αGal IgE and IgG, with a discrete impact on the total level of mouse IgE and IgG2b, respectively. However, since the sensitization process was conducted with SGE, we hypothesize that, together with the anti-αGal, other IgE and IgG2b specificities may be induced too. That is why the induction process impacted the total levels of IgE and IgG2b antibodies. Conversely, the treatment with DP1000-RA0118 was specific and only removed the anti-αGal antibodies. Interestingly, total circulating IgM was reduced after DP1000-RA0118 treatment, likely due to the significant contribution of anti-αGal to the circulating IgM repertoire of GalT-KO mice (15).

Finally, glycopolymers with DP<1000 have shown a high capacity to inhibit anti-αGal IgM and IgG *in vitro* but have been entirely ineffective *in vivo* (53). However, there is no data regarding the impact of DP on anti-αGal IgE inhibition. In our research, we found that DP100-RA0127 and DP100-RA0134 (100 µg/mL) showed high IgE-inhibition activity (>75%), with reduced IgG inhibition (<25%). This IgE isotype-dependent inhibition might be helpful for IgE-mediated diseases. Despite the *in vivo* administration of DP1000 αGal-conjugates has shown to be safe in primates and rodents (53), the impact of a lower DP on safety has not been evaluated so far. The polysaccharide chain length affects the immunogenicity of glycan-conjugated vaccines (87). In addition, the polysaccharide hapten size is critical in the immune response to carbohydrate vaccines (88). Indeed, reducing polysaccharide chain lengths improved vaccine immunogenicity (89, 90). Our experience developing monoclonal antibodies using Poly-L-lysine-OVA conjugate as the immunogen shows the poor immunogenicity of DP1000-Poly-L-lysine. Currently, we are improving the immunogenicity of Poly-L-lysine–OVA conjugates by reducing the length of the poly-L-lysine backbone (data not shown). With all these findings, we might expect higher toxicities associated with DP100-glycopolymers than DP1000. Therefore, before studying the efficacy of DP100-αGal-glycopolymers in the selective *in vivo* removal of IgE over the other anti-αGal isotypes, we consider conducting immunogenicity studies with DP100 crucial to rule out possible safety concerns.

Although the results presented here are promising, they have some limitations. First, the primary objective of this work was to develop a model of αGal-sensitization in mice; however, a more robust model of clinical AGS needs to be addressed to study the clinical impact of DP1000-RA0118 administration. Second, the most promising compounds (including DP100-RA0127 and DP100-RA0134) will need to be tested for their ability to elicit B cell hyporesponsiveness in GalT-KO mice sensitized to αGal. Finally, according to some epidemiological studies, anti-αGal, mainly IgM, may play a protective role against protozoan infections (19–21). Therefore, the potential benefit of having these induced antibodies in regions endemic for protozoan infections must be considered in case our therapy is implemented to treat any disease mediated by anti-αGal IgE. Nevertheless, it is essential to clarify that our therapy produced an immediate but transient inhibition of anti-αGal antibodies. More importantly, our data suggest that it is possible to get a differential anti-αGal isotype inhibition as a function of the length of the poly-L-lysine backbone and total residues of αGal exposure in the polymers.

The present work confirmed that hard ticks’ SGE elements are responsible for the induction of anti-αGal IgE antibodies. We postulate that the αGal sensitization mechanism may go through an “atypical” Th2-response (hypersensitivity type-I), which primarily led to IgG2b and IgE production in GalT-KO mice. Due to the high affinity showed by anti-αGal IgE antibodies for the assessed polymeric αGal-glycoconjugates, we hypothesize that the anti-αGal IgE in sensitized patients might come from IgG-switched B cells, unlike the classic atopic sensitization to pollen and mite allergens where IgE come from naive B cells. We demonstrated the potentiality of poly-L-lysine-based αGal-glycoconjugates for treating allergic disorders mediated by anti-αGal IgE antibodies. As AGS is spreading due to the expansion and changes of hard ticks’ habitats, the immunotherapy concept presented here, based on the selective removal of induced anti-αGal IgE antibodies with poly-L-lysine αGal-glycoconjugates, may provide a clinical solution to this disorder.

## Data Availability Statement

The raw data supporting the conclusions of this article will be made available by the authors, without undue reservation.

## Ethics Statement

The studies involving human participants were reviewed and approved by Clinical Research Committee of the Hospital Universitari de Bellvitge (PR212/17). The patients/participants provided their written informed consent to participate in this study. The animal study was reviewed and approved by ethics committee for animal experimentation of Bellvitge Biomedical Research Institute (IDIBELL). Serum samples from αGal-sensitized subjects (PT, n=13) were selected from previous epidemiological studies (59), where anti-αGal IgE prevalence of anti-αGal IgE in individuals with acute urticaria or anaphylaxis from different geographical areas of Spain was studied.

## Author Contributions

SO-A contributed to the experimental design, performed the experimental work related to animal samples collection, ELISA, and white blood cell profiling, and participated in the manuscript drafting. DB-G designed all the experimental work, coordinated the study, made a substantial contribution to data management and analysis, and wrote the body of the manuscript. RA performed the tick saliva extraction and contributed to the manuscript drafting. YF-A performed the experimental work related to ELISA and white blood cell profiling. BG and ML-H provided the clinical samples and significantly contributed to manuscript drafting. AG-P provided technical insights regarding the preliminary preparation of salivary gland extract and contributed during the manuscript drafting. NB and AT participated in synthetic conjugation strategy and contributed during manuscript drafting. RM significantly contributed to the manuscript conception and drafting. All authors contributed to the article and approved the submitted version.

## Conflict of Interest

SO-A, DB-G, and YF-A are employees of RemAb Therapeutics SL. DB-G and RM are shareholders of RemAb Therapeutics SL. SO-A and DB-G hold a patent on new glycoconjugates and medical uses thereof.

The remaining authors declare that the research was conducted in the absence of any commercial or financial relationships that could be construed as a potential conflict of interest.

The authors declare that this study received funding from RemAb Therapeutics SL. The funder had the following involvement in the study: design and complete execution, data generation, interpretation and graphing, article writing, edition, and submission.

## Publisher’s Note

All claims expressed in this article are solely those of the authors and do not necessarily represent those of their affiliated organizations, or those of the publisher, the editors and the reviewers. Any product that may be evaluated in this article, or claim that may be made by its manufacturer, is not guaranteed or endorsed by the publisher.
