# Supplementary material for: Poly-L-Lysine-Based αGal-Glycoconjugates for Treating Anti-αGal IgE-Mediated Diseases
Source: Front Immunol. 2022 Mar 31;13:873019. doi: 10.3389/fimmu.2022.873019 (PMC9009260; doi:10.3389/fimmu.2022.873019)
Supplement: Supplementary file 1 [file DataSheet_1.docx]

# Supplementary Figures

Intensity (Arbitrary units)


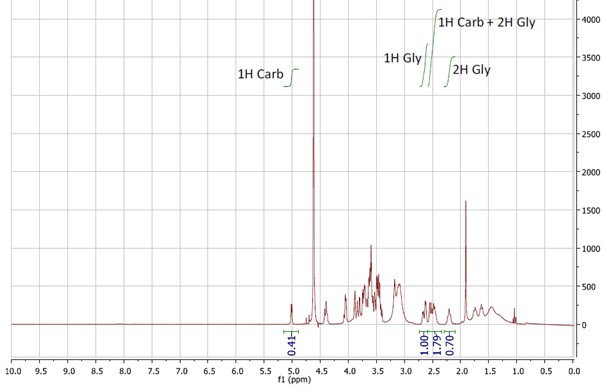


**Figure S1.** **^1^H NMR spectrum of GAS914 (Novartis) in D_2_O (30 ºC, 300 MHz).** The ratio of αGal to poly-L-lysine backbone was calculated from the integration of the αGal trisaccharide (Carb) and glycerol (Gly) residues at 5.2 ppm and 2.8 ppm in ^1^H NMR spectra, respectively.


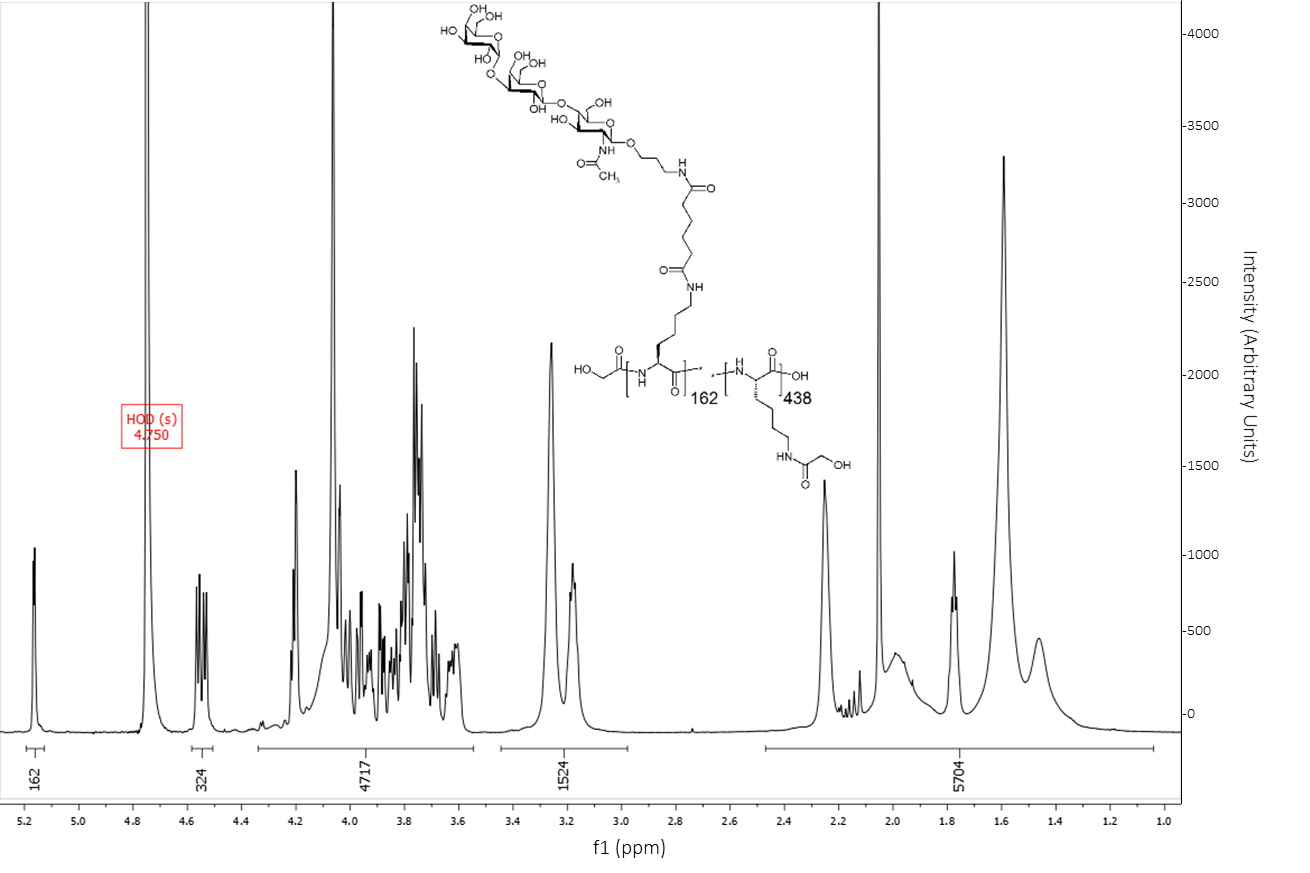


**Figure S2.** **Example of analysis of αGal trisaccharide load (% of substituted lysines) in the glycopolymers by ^1^H NMR spectroscopy (D2O, 30 ºC, 700 MHz).** Peak integration for DP600-RA0127: δ 5.165 (d, 162H, J=3.9 Hz; 162 H-1 of Galα), 4.750 (s, HOD), 4.561 and 4.535 (s, 162H and 162H, J=7.8 Hz and J=7.6 Hz; 162 H-1 of Galβ and 162 H-1 of GlcNAcβ), 4.330-3.604 (4716H; 600H of α-CH of Lys, 2916H of 162 αGal trisaccharide, 324 H of OCH_2_ of spacer and 876H of 438 C(O)CH_2_O), 3.260 and 3.180 (m, 1524H; 1200H of ε-CH_2_ of Lys and 324H of 126 CH_2_N of spacer), 2.249-1.463 (5706H; 3600H of β-, γ- and δ-CH_2_ of Lys, 486H of NC(O)CH_3_ of 162 GlcNAc, 324H of 162 CH_2_ of spacer and 1296H of 162 CH_2_CH_2_CH_2_CH_2_ adipic residues) ppm.


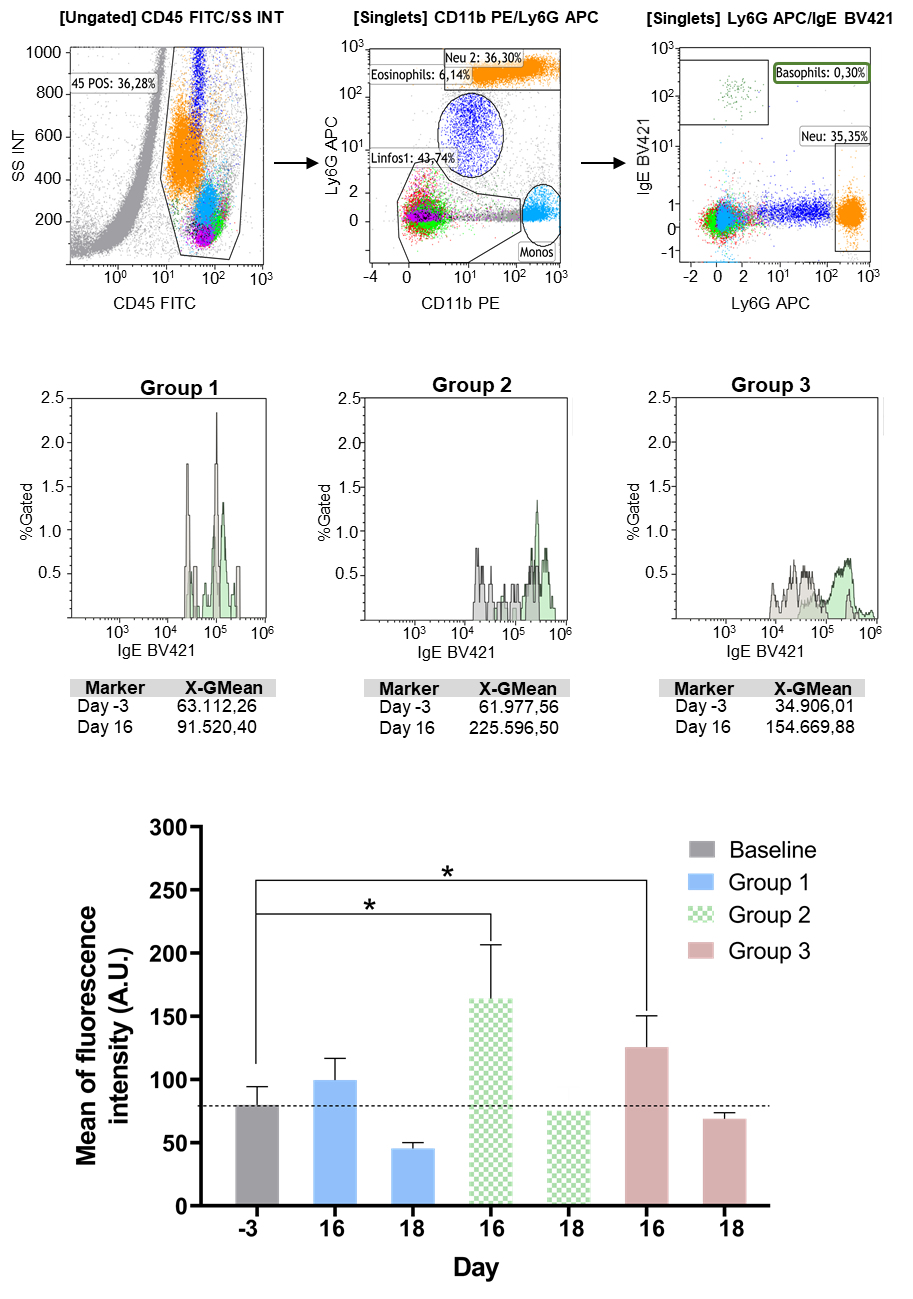


**B**

**C**

**A**

**Figure S3.** **Analysis of basophil population by Flow Cytometry**. αGal sensitization in GalT-KO mice with tick salivary gland extract induced a significant increase in fluorescence intensity for basophils on day 16 for Groups 2 and 3 compared to control Group 1. **(A)** Representative example of gating strategy to define basophil population. Events collected from fresh blood mouse samples were displayed in a CD45 vs. side scatter intensity (SS INT) plot to discard debris and define a total WBC population. Basophils were gated using rat anti-mouse Ly6G-APC and IgE-BV421, a monoclonal antibody that reacts to the IgE bound to Fcε receptors on basophils. **(B)** Geometric mean of fluorescence intensity for basophil population of representative samples from different experimental groups. **(C)** The columns represent the average geometric fluorescence intensity (± SEM) for the gated basophil population for the different experimental groups and days (n=6). Signal on day -3 (baseline conditions, grey) corresponds to the average of the geometric fluorescence intensity (± SEM) of all the mice involved in the study (n=18). FACS determination recorded 150,000 total events, of which about 50,000 were CD45 positive. Data were analyzed using KALUZA software (Beckman Coulter, CA, USA). Mann Whitney test analysis was performed to compare fluorescence between baseline vs. day 16 (*: p<0.05; **: p<0.01; ***: p<0.001; ****: p<0.0001).

# Supplementary Tables

Table S1. List of Poly-L-lysine glycoconjugates with different degrees of polymerization (DP) and αGal trisaccharide loads (9, 12, 18, 27, and 34%).

| **Name** | **PLys DP** | **αGal load** | **Total αGal residues** |
| --- | --- | --- | --- |
| DP100-RA0109 | 100 | 9% | 9 |
| DP600-RA0109 | 600 | 9% | 54 |
| DP1000-RA0109 | 1000 | 9% | 90 |
| DP100-RA0112 | 100 | 12% | 12 |
| DP600-RA0112 | 600 | 12% | 72 |
| DP1000-RA0112 | 1000 | 12% | 120 |
| DP100-RA0118 | 100 | 18% | 18 |
| DP600-RA0118 | 600 | 18% | 108 |
| DP1000-RA0118 | 1000 | 18% | 180 |
| DP100-RA0127 | 100 | 27% | 27 |
| DP600-RA0127 | 600 | 27% | 162 |
| DP1000-RA0127 | 1000 | 27% | 270 |
| DP100-RA0134 | 100 | 34% | 34 |
| DP600-RA0134 | 600 | 34% | 204 |

Table S2. Antibody panel used in Flow Cytometry analyzes.

| **Detector** | **Band Pass Filter** | **Fluorochromes** | **Antibody** |
| --- | --- | --- | --- |
| FL1 | 525/40 | FITC | Rat Anti-Mouse CD45 |
| FL2 | 575/30 | PE | Rat Anti-CD11b |
| FL3 | 620/30 | PE-CF594 | Rat Anti-Mouse CD8a |
| FL4 | 695/40 | BB700 | Rat Anti-Mouse CD193 |
| FL5 | 755LP | PE-Cy7 | Rat Anti-Mouse CD19 |
| FL6 | 660/20 | APC | Rat Anti-Mouse Ly-6G |
| FL7 | 725/20 | APC-R700 | Rat Anti-Mouse CD5 |
| FL8 | 755LP | PC-H7 | Rat Anti-Mouse CD4 |
| FL9 | 450/50 | BV421 | Rat Anti-Mouse IgE |
| FL10 | 550/40 | V500 | Syrian Hamster anti-Mouse CD3e |
